# Steroids Therapy in Patients With Severe COVID-19: Association With Decreasing of Pneumonia Fibrotic Tissue Volume

**DOI:** 10.3389/fmed.2022.907727

**Published:** 2022-07-14

**Authors:** Jin-wei He, Ying Su, Ze-song Qiu, Jiang-jie Wu, Jun Chen, Zhe Luo, Yuyao Zhang

**Affiliations:** ^1^School of Information Science and Technology, ShanghaiTech University, Shanghai, China; ^2^Department of Critical Care Medicine, Zhongshan Hospital, Fudan University, Shanghai, China; ^3^Department of Radiology, Renmin Hospital of Wuhan University, Wuhan, China; ^4^iHuman Institute, ShanghaiTech University, Shanghai, China

**Keywords:** COVID-19, chest-CT, steroids, quantitative analysis, pneumonia treatment

## Abstract

**Background:**

We use longitudinal chest CT images to explore the effect of steroids therapy in COVID-19 pneumonia which caused pulmonary lesion progression.

**Materials and Methods:**

We retrospectively enrolled 78 patients with severe to critical COVID-19 pneumonia, among which 25 patients (32.1%) who received steroid therapy. Patients were further divided into two groups with severe and significant-severe illness based on clinical symptoms. Serial longitudinal chest CT scans were performed for each patient. Lung tissue was segmented into the five lung lobes and mapped into the five pulmonary tissue type categories based on Hounsfield unit value. The volume changes of normal tissue and pneumonia fibrotic tissue in the entire lung and each five lung lobes were the primary outcomes. In addition, this study calculated the changing percentage of tissue volume relative to baseline value to directly demonstrate the disease progress.

**Results:**

Steroid therapy was associated with the decrease of pneumonia fibrotic tissue (PFT) volume proportion. For example, after four CT cycles of treatment, the volume reduction percentage of PFT in the entire lung was −59.79[±12.4]% for the steroid-treated patients with severe illness, and its *p*-value was 0.000 compared to that (−27.54[±85.81]%) in non-steroid-treated ones. However, for the patient with a significant-severe illness, PFT reduction in steroid-treated patients was −41.92[±52.26]%, showing a 0.275 *p*-value compared to −37.18[±76.49]% in non-steroid-treated ones. The PFT evolution analysis in different lung lobes indicated consistent findings as well.

**Conclusion:**

Steroid therapy showed a positive effect on the COVID-19 recovery, and its effect was related to the disease severity.

## Introduction

The coronavirus disease 2019 (COVID-19) pandemic has posed a great challenge to the world’s healthcare security (1). Steroids have been applied in pneumonia treatment for a long time, and they have shown positive efficacy during the treatment of SARS (2) and MERS (3). Therefore, the latest WHO treatment opinion strongly recommends systemic steroids for severe and critically ill patients with COVID-19 (4). However, the steroids’ use in pneumonia is still controversial for the absence of specific evidence to systematically expound its effect (5). Based on some literature, steroid therapy could suppress the inflammatory storm in the patient’s body and prevent the patient from suffering bad outcomes because of the excessive inflammatory response (6, 7). However, the use of steroids may also decrease the ability of the patient’s organism to clear the virus while prolonging the time of virus clearance (8, 9). Therefore, the effect of steroid therapy on patients with COVID-19 is in urgent need to be evaluated.

Beyond clinical and biological variables, computed tomography (CT) scans of patients with pneumonia carry salient disease information (10, 11). Therefore, CT has been proposed as an ancillary approach for screening individuals with suspected COVID-19 pneumonia during the epidemic period and monitoring treatment response according to the dynamic radiological changes (12–14). Traditional CT image analysis methods such as manual or semiquantitative assessments rely on the previous experience of radiologists, and these methods are subjective, time-consuming, and lack interobserver consistency (15, 16). As a fast, accurate and reproducible analytical tool, quantitative CT image analysis has been increasingly implemented in pulmonary diseases to extract objective data that can aid in lesion characterization and quantification (17, 18).

In this work, we aim to study the effect of steroids on pulmonary lesion progression. To achieve a well-controlled comparison between patients with and without steroid therapy, we only consider the monitored disease progression during hospitalization. Specially, we retrospectively enrolled a number of 78 patients with severe to critical COVID-19 pneumonia, among which 25 patients (32.1%) received steroid therapy. Serial longitudinal chest CT scans were performed for each patient during hospitalization. Through the quantitative analysis method, the volume changes of normal tissue and pneumonia fibrotic tissue (PFT) in the entire lung and each five lung segmentation lobes were the primary outcomes.

The major findings in this article are as follows:

1. Steroid therapy could promote the recovery of patients with COVID-19 with severe illness.
2. Steroid therapy could promote the recovery of patients with COVID-19 with significant-severe/critical illness, but its effect still needs further research.
3. CT quantitative analysis results could be used in disease severity evaluation and may be an important reference for deciding the use of steroids.

## Patients and Methods

### Patient Involvement

We retrospectively involved patients consecutively admitted from 31 January to 17 February in the east campus of the Renmin Hospital of Wuhan University. The patients were confirmed of COVID-19 based on the detection of SARS-CoV-2 nucleic acid by real-time RT–PCR assay. The patient inclusion criteria are as follows: (1) age ≥18 years, (2) patients with severe or critical COVID-19, and (3) patients with at least three CT scans during the hospital stay. To ensure that the lesions in the quantitative analysis were caused by COVID-19, we excluded the patient with hematological or solid malignancies. In addition, to avoid the patient’s immune system being affected by the drugs administered before COVID-19 treatment, patients who went through systemic steroids or immunosuppressive therapy in the previous 6 weeks before admission were also excluded.

This study was approved by the Ethics Committee of the Wuhan University People’s Hospital and was conducted in accordance with the Declaration of Helsinki. The Ethics Committee waived written informed consent in the setting of the Wuhan COVID-19 crisis.

### Clinical Trials

Among the total enrollment of 78 patients with severe to critical COVID-19 pneumonia, 25 patients (32.1%) received steroid therapy treatment during hospitalization. All the steroid therapies were initiated at the time of admission at the discretion of attending physicians based on clinical symptoms and CT images. According to previous experience (19–22), intravenous methylprednisolone at a dose of 1.0–1.5 mg/kg every 12 h was initiated for 5 days or until oxygen saturation improved, followed by gradual tapering by 0.5 mg/kg every 3–5 days.

For all the patients, other therapeutic interventions were performed following the sixth edition of the Guidelines on the Diagnosis and Treatment of COVID-19 published by the National Health Commission of China.

### Patient Category

For a more comprehensive study, we further divided 78 cases into four groups to explore disease evolution for different levels of severities. The COVID-19 disease severity was evaluated according to the WHO interim guidance (4) or the sixth version of Chinese national guidelines on the diagnosis and treatment for COVID-19 (23). Patients were considered as a severe condition if they met any of the following conditions: respiratory distress and a respiratory rate >30 times per minute; oxygen saturation on room air at rest <93%, and partial arterial oxygen pressure (PaO_2_)/fraction of inspiration oxygen (FiO_2_) ≤300 mmHg. Patients were considered critical if they met any of the following conditions: respiratory failure requiring mechanical ventilation, shock, and organ dysfunction requiring ICU management. In this work, patients who met one clinical symptom listed above are considered severe cases. And those who met more than one clinical symptom are considered significant-severe cases. The patients with critical conditions are considered as significant-severe cases as well.

Therefore, the four groups are the non-steroids-treated group (NST-group) with severe (NST-S: 46) cases and significant-severe (NST-SS: 7 cases) illness; and the steroids-treated group (ST-group) with severe (ST-S: 12 cases) and significant-severe (ST-SS: 13 cases) illness. The clinic characteristics of each group are shown in Table 1. It is worth noticing that the ST group involves more SS cases, because, in the treatment plan, physicians tend to use steroid therapy for more severe cases.

**TABLE 1:** Clinical characteristics of all the patients.

Patient category	NST-S	ST-S	*P*-value	NST-SS	ST-SS	*P*-value
Patient number	46	12		7	13	
Mean CT intensity on admission (HU)	−803.45 [66.84]	−714.76 [85.06]	0.007	−710.74 [171.04]	−528.70 [201.56]	0.063
Average age (years)	60 [14]	53 [10]	0.049	63 [14]	69 [9]	0.133
Average hospital stay (Days)	28 [15]	33 [5]	0.012	36 [14]	41 [11]	0.302
Gender (male), n (%)	23 (50)	4 (33.33)	0.348	4 (57.14)	8 (61.54)	1.000
Fever, n (%)	37 (80.43)	12 (100)	0.181	6 (85.71)	13 (100)	0.350
Breathing disorder, n (%)	18 (39.13)	5 (41.67)	1.000	7 (100)	12 (92.31)	1.000
Cough, n (%)	34 (73.91)	8 (66.67)	0.720	5 (71.43)	9 (69.23)	1.000
Expectoration, n (%)	8 (17.40)	1 (8.33)	0.668	0 (0.00)	2 (15.38)	0.521
Nausea or vomiting, n (%)	7 (15.22)	3 (25.00)	0.417	1 (14.29)	2 (15.38)	1.000
Diarrhea, n (%)	6 (13.04)	4 (33.33)	0.191	1 (14.29)	3 (23.07)	1.000
Fatigue, n (%)	38 (82.61)	10 (83.33)	1.000	7 (100.00)	11 (84.62)	0.521
Headache, n (%)	3 (6.52)	1 (8.33)	1.000	0 (0.00)	0 (0.00)	1.000
Muscle or joint pain, n (%)	3 (6.52)	1 (8.33)	1.000	1 (14.29)	2 (15.38)	0.917

*For continuous variables, normally distributed data were shown as mean [SD], and non-normally distributed data were shown as median [IQR]. Categorical variables were shown as proportional number (%). SD, standard deviation; IQR, interquartile range; NST-S, non-steroids-treated group with severe illness; ST-S, steroids-treated group with severe illness; NST-SS, non-steroids-treated group with significant-severe illness; ST-SS, steroids-treated group with significant-severe illness; M/F, Male/Female.*

### Chest Computed Tomography Protocol

All the patients underwent serial chest CT scans during hospitalization to monitor disease progression. The first scan was on admission, and the followed-up scans were conducted during their hospitalization with an average time interval of 6[1] days in-between each scanning. All the CT scans were performed using a single inspiratory phase on a commercial multidetector CT scanner (GE Optima CT680). To minimize motion artifacts, CT images were acquired in a single breath, using standard lung scanning settings as follow: 120 kV and automatic tube current (180–400 mA); iterative primary reconstruction technique; and 64 mm detector: rotation time = 0.35 s: partial-thickness = 5 mm; alloy = 0.625 mm; spacing = 1.5 mm; and matrix = 512 × 512.

### Chest Computed Tomography Image Processing and Quantification

#### Lung Extraction and Segmentation

**FIGURE 1:**
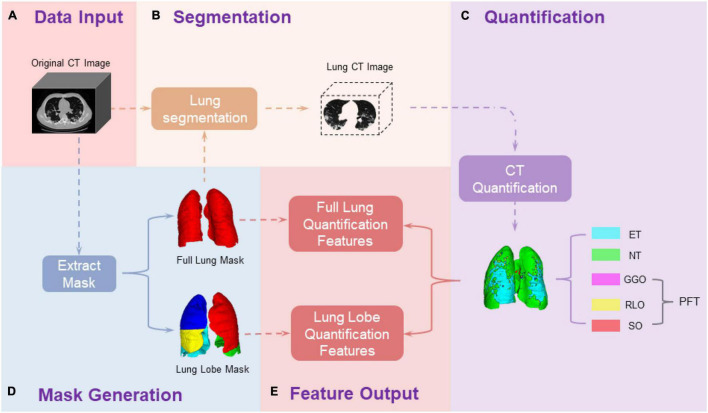
Pipeline of chest CT image processing and quantification. **(A)** Collect the original CT images of patients; **(D)** Generate the full lung mask and lung lobe mask through a pre-trained neural network; **(B)** Extract the CT data of ROI from the original CT image based on the ROI masks; **(C)** Quantify the ROI CT data based on the HU values; **(E)** calculate the data outcomes. The primary outcomes are different tissue volume proportion in the entire lung region and each lung lobe along longitudinal CT time points, which are generated in **(E)** Feature Output step as: $\%Tissue_{C\,T_{i}}=\frac{T\,i\,s\,s\,u\,e\,v\,o\,l\,u\,m\,e\,a\,t\,C\,T_{i}\,i\,n\,R\,O\,I}{T\,o\,t\,a\,l\,v\,o\,l\,u\,m\,e\,o\,f\,R\,O\,I\,a\,t\,C\,T_{i}}$, where *CT*_*i*_ = {*CT*_0_,⋯,*CT*_4_}, *Tissue* = {*ET*, *NT*, *GGO*, *RLO*, *SO*}, and *ROI* {fulllung, LUL, LLL, RUL, RML, RLL}. ET, emhpysema lung tissue; NT, normal lung tissue; PFT, pneumonia fibrotic tissue; GGO, ground-glass opacity; RLO, reticular and linear opacification; SO, consolidations.

As demonstrated in Figure 1, we first conducted lung segmentation from the chest CT image using a pretrained lung segmentation neural network, especially proposed for COVID-19-infected lungs (24), generating both whole lung mask and lung lobe labels. The generated lung lobe labels contained the left upper lobe (LUL), the left lower lobe (LLL), the right upper lobe (RUL), the right middle lobe (RML), and the right lower lobe (RLL), respectively.

#### Pulmonary-Lesion-Level Quantification

We assessed the pulmonary lesion level in each lung lobe, referring to the CT HU range. As reported in several studies (25–27), pulmonary tissue can be mapping into: emphysema lung tissue (ET) [−1,024, −950] HU, normal lung tissue (NT) [−950, −700] HU, ground-glass opacity (GGO) [−700, −534] HU, reticular and linear opacification (RLO) [−534, −188] HU, and consolidations (SO) [−188, 300] HU. The lung tissue, whose CT HU value was greater than −700 HU, which included GGO, RLO, and SO, was defined together as pneumonia fibrotic tissue (PFT).

#### Tissue Volume Proportion Evolution Along Different Computed Tomography Scan Time

For each patient, the longitudinal extracted lung images were denoted as *CT*_0_, *CT*_1_⋯,* and CT*_*n*_, where *CT*_0_ was the baseline CT scan at admission;*CT*_1_⋯, *and CT*_*n*_, were the follow-up scans, the number *n* depended on the disease duration of the patient.

We further assessed the effect of steroids on the evolution of COVID-19 pneumonia progression by computing volume proportion in each different level of pulmonary tissue, alternations on tissue percentage %*Tissue* = %*ET*, %*NT*, %*GGO*, %*RLO*, %*SO*. Specifically, the percentage of different lesion levels in the entire lung and each of the five lung lobes (LUL, LLL, RUL, RML, and RLL) were computed, respectively, as the following equations: $\%Tissue_{C\,T_{i}}=\frac{T\,i\,s\,s\,u\,e\,v\,o\,l\,u\,m\,e\,a\,t\,C\,T_{i}\,i\,n\,R\,O\,I}{T\,o\,t\,a\,l\,v\,o\,l\,u\,m\,e\,o\,f\,R\,O\,I\,a\,t\,C\,T_{i}}$, where *Tissue* = {*ET*, *NT*, *GGO*, *RLO*, and *SO*}, and *ROI* = {fulllung, LUL, LLL, RUL, RML, and RLL}. For example, in the LUL lobe, $\%ET_{C\,T_{0}}\frac{E\,T\,v\,o\,l\,u\,m\,e\,a\,t\,C\,T_{0}\,i\,n\,L\,U\,L}{T\,o\,t\,a\,l\,v\,o\,l\,u\,m\,e\,o\,f\,L\,U\,L\,a\,t\,C\,T_{0}}$.

### Data Calculation

#### Outcomes Assessment

We used the %*Tissue* as the primary outcome to monitor COVID-19 progression. Thus, the increase of %*NT* and the decrease of %*PFT* (%*GGO*, %*RLO*, and %*SO*) indicated the alleviation of pulmonary infections, which presented the improvement of disease condition.

The secondary outcomes were the baseline normalized (BN) tissue volume, defined as: *Tissue*_*BN*_*CT*_*i*__, (*ET*_*BN*_*CT*_*i*__, *NT*_*BN*_*CT*_*i*__, *ET*_*PFT*_*CT*_*i*__,*GGO*_*BN*_*CT*_*i*__, *RLO*_*RLO*_*CT*_*i*__, and *ET*_*SO*_*CT*_*i*__). We normalized the tissue volume of each type by dividing its volume at *CT*_0_:$T\,i\,s\,s\,u\,e_{B\,N\,\_\,C\,T_{i}}=\frac{T\,i\,s\,s\,u\,e\,v\,o\,l\,u\,m\,e\,a\,t\,C\,T_{i}\,i\,n\,R\,O\,I}{T\,i\,s\,s\,u\,e\,v\,o\,l\,u\,m\,e\,o\,f\,R\,O\,I\,a\,t\,C\,T_{0}}$. After normalization, the values of *Tissue*_*BN*_*CT*_0__ were all equal to 1. The value of *Tissue*_*BN*_*CT*_*i*__ above 1 indicated that the tissue volume increased over time. In a way, *Tissue*_*BN*_*CT*_*i*__ could reflect the rate of recovery. The higher value of *NT*_*BN*_*CT*_ and the lower value *PFT*_*BN*_*CT*_*i*__ both reflected an improvement of disease condition.

#### Statistical Method

We conducted significant tests to confirm the statistically significant difference between the patients’ disease progression with and without steroid therapy. Our null hypothesis was that there was no significant variation between the disease progression of the two types of treatment. While the *p*-value was less than 0.05, we considered the null hypothesis false and therefore existed a statistical difference between tested patient groups. The clinical characteristics are presented in Table 1. For continuous variables, normally distributed data were presented as mean with SD, and non-normally distributed data were presented as median with interquartile range (IQR). The Student’s *t*-test was used for normally distributed continuous variables, and the Mann–Whitney *U* test was used for non-normally distributed continuous variables. For serial quantitative CT parameters (e.g., %*Tissue* at different time points), we tested differences between two groups over time by using repeated-measures ANOVA with no imputation for missing values. In addition, we conducted a significant test of each tissue type in volume percentage for the entire lung and each five lung lobes. A two-sided *p*-value less than 0.05 was considered to be statistically significant.

## Results

### Patient Characteristics

From 31 January to 17 February, 111 patients with COVID-19 having severe or critical illness were admitted and screened. Among these patients, 33 were excluded, including 30 patients with less than 3 serial CT scans, two patients receiving systemic corticosteroids or immunosuppressive therapy in the previous 6 weeks, and one patient with solid malignancy. And finally, there were 78 patients with COVID-19 (53 patients without steroid therapy and 25 patients with steroid therapy) with severe and significant-severe and critical illness were included in the analyses. Furthermore, they were divided into four groups: NST-S (46 cases), NST-SS (7 cases), ST-S (12 cases), and ST-SS (13 cases).

Table 1 presents the baseline clinical characteristics of the patient in these four groups. Fever, fatigue, and cough were the three most common symptoms. The baseline characteristics were similar (*p*-value > 0.05) for the patients with the same disease severity. Their vital signs and the PaO_2_/FiO_2_ ratio on admission were also similar. In each trial, the patients treated with steroids presented higher mean CT intensity value at baseline moment (ST-S: −714.76[±85.06] HU vs. NST-S: −803.45[±66.84] HU, *p*-value = 0.007 and ST-SS: −528.70[±201.56] HU vs. NST-SS: −710.74[±171.04] HU, *p*-value = 0.063). For average hospital stay, patients treated with steroids had a relatively (not significantly) longer hospital stay as well (ST-S: 33[±5] days vs. NST-S: 28[±15] days, *p*-value = 0.012 and ST-SS: 41[±11] days vs. NST-SS: 36[±16] days, *p*-value = 0.302).

### Computed Tomography Quantitative Result Proportional Changes Over Time

This study quantitatively evaluated proportional volume variations and normalized tissue volume of different tissue types in the severe group and significant-severe group using different therapy treatment. The evaluations were performed in the entire lung and five different lung lobes, respectively.

For the trial of severely ill patients, at *CT*_0_ moment (baseline moment at admission) in Supplementary Table 1, %*NT* in the ST-S group was significantly lower than that in the NST-S group (i.e., ST-S: 68.56% vs. NST-S: 84.6%, *p*-value = 0.01 in the full lung), and %*PFT* were much higher (i.e., ST-S: 31.06% vs. NST-S: 13.67%, *p*-value = 0.01 in the full lung). Moreover, all five %*Tissue* values existed statistical difference (*p*-value < 0.05) between the NST-S group and ST-S group in the entire lung, and especially in lobe LLL, RML, and RLL. However, most of these differences went away after treatment of four CT cycles (on average 4 weeks). As indicated in Supplementary Table 1 at *CT*_4_, the %*NT* and %*PFT* percentage values in the ST-S group became much closer to those in the NST-S group, which were observed in all ROIs.

For the trial of significant-severe ill patients in Supplementary Table 2, at *CT*_0_%*SO* in ST-SS group of the full lung was significantly higher than that in the NST-SS group (i.e., ST-S: 14.13% vs. NST-S: 6.89%, *p*-value = 0.08 in the full lung). The statistical differences at *CT*_0_ of the full lung were mainly brought by lobe LUL. As shown in Supplementary Table 2, all statistical differences disappeared after four cycles of treatment, and %*SO* became close in these two groups (ST-SS: 3.87% vs. NST-SS: 2.50% *p*-value = 0.127 in the full lung). However, %*PFT* in the ST-SS group was still 10% higher than that in the NST-SS group (i.e., 36.24 vs. 20.02% in the full lung *p*-value = 0.106) at *CT*_4_.

We plot the variations of *Tissue*_*BN*_ (as defined in section “Outcomes Assessment”) as curves along with CT scanning time points in Supplementary Figure 1. In Part (A), in all ROIs, the ST-S curve is above the NST-S curve in *NT*_*BN*_ [indicating the increased volume of normal tissue (NT) compared with *CT*_0_] and below the NST-S curve in *PFT*_*BN*_ (demonstrating decreased volume of compromised tissue compared with *CT*_0_) during hospitalization. Similar decreasing curve trends in *GGO*_*BN*_ and *RLO*_*BN*_ of Part (B) can also be observed. Notably, the error bar of the ST-S curve is much smaller than that of the NST-S curve at *CT*_4_, presenting more similar illness level conditions among different patients in the ST-S group. For patients with significant-severe illness, in the full lung, the ST-SS curve is above NST-SS curve in *NT*_*BN*_ and below NST-SS curve in *PFT*_*BN*_ (especially in *RLO*_*BN*_ and *SO*_*BN*_), both indicating more increased normal tissue and more decreased compromised tissue for steroid therapy treated patients. However, similar results do not arise in all five lung lobes. Especially for the *GGO*_*BN*_, the ST-SS group presents an upward trend (*GGO*_*BN*_ 1) in LUL, LLL, and RLL, which is not observed in the NST-SS group.

Table 2 shows the change of *Tissue*_*BN*_ (△%*Tissue*_*BN*_), from *CT*_0_ to *CT*_4_: △%*Tissue*_*BN*_ (*Tissue*_*BN*_*CT*_4__−*Tissue*_*BN*_*CT*_0__)^*^100% (*Tissue*_*BN*_*CT*_4__−1)^*^100%. After four CT cycles of treatment, for the patients with severe illness, the changes of *NT*_*BN*_ and *PFT*_*BN*_ showed a statistical difference (*p*-value < 0.05) in the full lung and all the five lung lobes between the patients with and without steroid treating. The steroid-treated patients showed a higher proportion of increase in *NT*_*BN*_ and decrease in *PFT*_*BN*_ (i.e., for △%*NT*_*BN*_, ST-S: 26.31[±3.09] vs. NST-S: 1.4[±19.26], *p*-value = 0.002 and for △%*PFT*_*BN*_, ST-S: −59.79[±12.4] vs. NST-S: −27.54 [±85.81], *p*-value = 0.000). For the patients with significant-severe illness, there were no statistical differences of the variation △%*PFT*_*BN*_ in all ROIs between the patients with and without steroids (ST-SS: −41.92[±52.26] vs. NST-SS: −37.18[±76.49], *p*-value = 0.275 in the full lung). And the statistical difference of the △%*NT*_*BN*_ only existed in the full lung and left upper lobe (ST-SS: −41.92[±52.26] vs. NST-SS: 16.23[±42.76], *p*-value = 0.029 in the full lung).

**TABLE 2:** The changes of normalized CT quantitative results after 5-CT-cycle treating.

Lung region	Patient category	NST-S	ST-S	*P*-value	NST-SS	ST-SS	*P*-value
	Patient number	16	9		6	10	
Full lung	△%*ET*_*BN*_	175.22 [313.04]	206.25 [364.58]	0.222	220.16 [520.97]	286.15 [352.31]	0.478
	△%*NT*_*BN*_	1.4 [19.26]	26.31 [3.09]	0.002	16.23 [42.76]	64.4 [75.52]	0.029
	△%*PFT*_*BN*_	−27.54 [85.81]	−59.79 [12.4]	0.000	−37.18 [76.49]	−41.92 [52.26]	0.275
	△%*GGO*_*BN*_	−18.39 [98.73]	−42.13 [17.97]	0.002	−16.93 [90.32]	28.08 [69.34]	0.435
	△%*RLO*_*BN*_	−26.59 [98.31]	−65.66 [11.47]	0.000	−42.87 [73.13]	−49.46 [47.48]	0.275
	△%*SO*_*BN*_	−44.47 [84.74]	−61.3 [9.29]	0.009	−63.64 [39.91]	−72.61 [61.22]	0.478
Left upper lung	△%*ET*_*BN*_	197.76 [362.18]	273.02 [100.0]	0.161	225.88 [738.82]	296.81 [487.23]	0.435
	△%*NT*_*BN*_	−1.42 [13.47]	10.12 [2.12]	0.005	7.29 [32.99]	13.55 [38.21]	0.029
	△%*PFT*_*BN*_	−16.76 [42.18]	−49.36 [6.88]	0.001	−35.6 [100.9]	−22.99 [61.86]	0.393
	△%*GGO*_*BN*_	−20.02 [43.73]	−42.48 [9.51]	0.001	−39.91 [103.81]	55.85 [111.19]	0.352
	△%*RLO*_*BN*_	−14.67 [44.5]	−52.61 [7.23]	0.000	−31.03 [83.78]	−40.53 [46.75]	0.151
	△%*SO*_*BN*_	−36.4 [53.6]	−47.43 [14.86]	0.123	−28.61 [74.13]	−59.42 [55.62]	0.029
Left lower lung	△%*ET*_*BN*_	128.77 [315.09]	137.21 [576.74]	0.444	120.9 [317.91]	128.79 [136.36]	0.478
	△%*NT*_*BN*_	4.34 [23.32]	43.74 [7.67]	0.000	20.87 [53.29]	−0.74 [101.05]	0.352
	△%*PFT*_*BN*_	−36.96 [124.03]	−61.65 [13.59]	0.004	−32.57 [77.43]	−0.4 [115.27]	0.313
	△%*GGO*_*BN*_	−31.77 [165.25]	−39.18 [26.08]	0.112	−10.74 [65.43]	7.32 [77.98]	0.178
	△%*RLO*_*BN*_	−24.37 [114.6]	−71.61 [10.65]	0.000	−34.1 [116.02]	6.97 [154.24]	0.240
	△%*SO*_*BN*_	−51.05 [63.45]	−76.64 [11.59]	0.003	−57.29 [77.06]	−53.82 [101.23]	0.435
Right upper lung	△%*ET*_*BN*_	423.15 [711.11]	640.0 [410.0]	0.19	460.53 [773.68]	373.91 [793.48]	0.208
	△%*NT*_*BN*_	−1.91 [14.48]	10.47 [3.95]	0.004	35.29 [37.86]	10.98 [41.05]	0.151
	△%*PFT*_*BN*_	−6.4 [109.1]	−52.0 [6.75]	0.000	−60.94 [48.23]	−22.2 [82.15]	0.106
	△%*GGO*_*BN*_	−8.62 [112.21]	−44.28 [15.13]	0.000	−33.32 [81.97]	−5.3 [75.44]	0.393
	△%*RLO*_*BN*_	0.54 [111.11]	−51.26 [11.76]	0.000	−63.67 [38.4]	−38.63 [69.41]	0.106
	△%*SO*_*BN*_	−21.37 [91.45]	−56.22 [10.95]	0.008	−66.84 [48.21]	−64.7 [61.44]	0.478
Right middle lung	△%*ET*_*BN*_	120.93 [218.02]	164.52 [238.71]	0.123	114.6 [324.82]	291.84 [343.88]	0.275
	△%*NT*_*BN*_	−2.92 [10.85]	16.95 [2.82]	0.002	3.64 [29.49]	1.93 [31.55]	0.24
	△%*PFT*_*BN*_	−1.24 [88.49]	−60.35 [2.88]	0.000	−20.03 [130.57]	−8.02 [73.84]	0.393
	△%*GGO*_*BN*_	0.35 [99.53]	−56.62 [3.22]	0.000	−15.15 [153.44]	−7.77 [78.07]	0.352
	△%*RLO*_*BN*_	2.99 [77.31]	−60.04 [10.86]	0.000	−37.24 [96.06]	−15.78 [83.93]	0.478
	△%*SO*_*BN*_	−25.0 [40.77]	−42.35 [18.37]	0.022	−59.81 [20.56]	−41.3 [76.4]	0.313
Right lower lung	△%*ET*_*BN*_	210.38 [311.32]	278.26 [608.7]	0.399	262.86 [485.71]	140.2 [296.08]	0.106
	△%*NT*_*BN*_	5.3 [38.87]	59.9 [5.01]	0.000	24.73 [59.82]	5.16 [71.29]	0.178
	△%*PFT*_*BN*_	−29.94 [193.73]	−67.96 [8.57]	0.001	−30.67 [65.16]	−8.8 [71.44]	0.127
	△%*GGO*_*BN*_	−20.38 [155.99]	−45.34 [23.72]	0.015	1.12 [45.39]	33.1 [67.18]	0.178
	△%*RLO*_*BN*_	−33.28 [168.51]	−73.11 [6.45]	0.000	−32.24 [84.77]	−13.2 [87.95]	0.208
	△%*SO*_*BN*_	−36.36 [62.27]	−70.88 [8.59]	0.003	−66.92 [56.62]	−69.38 [77.82]	0.275

*For the change of CT quantitative results, normally distributed groups were shown as mean [SD], and non-normally distributed groups were shown as median [IQR]. CT quantitative results were expressed in the form of volume percentage related to the full lung and each lung lobe. SD, standard deviation; IQR, interquartile range; NST-S, non-steroids-treated group with severe illness; ST-S, steroids-treated group with severe illness; NST-SS, non-steroids-treated group with significant-severe illness; ST-SS, steroids-treated group with significant-severe illness; ET, emphysema lung tissue; NT, normal lung tissue; PFT, pneumonia fibrotic tissue; GGO, ground-glass opacity; RLO, reticular and linear opacification; SO, consolidations.*

## Discussion

The treatment of severe COVID-19 pneumonia plays an essential role in reducing mortality. An excessive inflammatory response can induce deleterious effects and lead to tissue damage mechanisms for patients under severe conditions (28). Steroids have been commonly used as an adjunctive therapy during serious infections for their immunomodulatory, though their clinical effect is controversial (29). Some previous research indicates that the proper use of steroids in severe COVID-19 may be beneficial but do not recommend in routine treatment (30, 31). And in this study, we explore the effect of steroid therapy COVID-19 treatment on patients with different pneumonia severities. To better monitor the effects of steroid therapy on patient recovery, we set some criteria to screen patients in this work. Especially only the survivors that recovered from the treatment were included and no one died during the treatment. To our best knowledge, this is the first study to further assess the effect of steroids on different severe COVID-19 levels. Our results show that the effect of steroids is related to the pneumonia severity and it shows a good effect in patients with severe illness.

Chest CT plays an important role in the diagnosing COVID-19 (32). Jiang et al. evaluated main CT features and semiquantitative scores representing disease severity among different clinical types (33). Their work indicated that CT images could accurately assess the severity of COVID-19 and help monitor the disease progression (33). However, semi-quantitative visual assessment highly relies on the experience of the observer. It is time-consuming, lacks reproducibility, and has interobserver and even intraobserver variability (15). For the longitudinal study in this work, semi-quantitative methods may significantly affect the accuracy and feasibility of the results. Recent studies state that the quantitative CT analysis method correlates well with semiquantitative method in disease severity evaluation (16, 34). The quantitative CT analysis method is efficient standardized, and the result is consistently highly reproducible (17, 35). It has been shown that quantitative CT analysis is an efficient tool to monitor the disease progress and evaluate the treatment effectiveness (18, 36, 37). In this work, we longitudinally assessed the progress of COVID-19 through the quantitative CT analysis method. The lung tissue, whose CT HU value was greater than −700 HU, was defined as pneumonia fibrotic tissue (PFT). The volume of PFT is the main index to evaluate disease severity. The decrease of %PFT and △%*PFT*_*BN*_ reflect an improvement of the disease condition.

In our study, steroid therapy shows a positive efficacy in accelerating the recovery of compromised lung tissue in patients with severe illness. In contrast, this effect still exists in patients with significant-severe illness but is limited. For patients with severe illness, the patients in the ST-S group recovered better and faster than those in the NST-S group. Their △%*PFT*_*BN*_ show a significant different distribution at *CT*_4_ (*p*-value = 0.00). However, for patients with significant-severe illness, though treated in four CT cycles, %*PFT* in ST-SS group patients decreases a lot, the △%*PFT*_*BN*_ in the ST-SS and NST-SS group do not demonstrate statistical differences (e.g., *p*-value = 0.275 in full lung). The limited efficacy shown in patients with significant-severe illness may be due to the insufficient number of patients. Some recent studies reporting positive effects on steroid treatment of COVID-19 (30, 31) might be more related to the patient group with severe illness. And the more specific outcomes of the steroids used in the patients with significant-severe illness still require further studies.

In addition, as shown in our study, the effect of steroids may associate more with the CT performance compared with the clinical symptoms. In this work, the disease severity is classified by clinical symptoms. But for patients with significant-severe illness, CT mean intensity at admission time are significant different between the NST-SS group and ST-SS group (*p*-value = 0.023). Patients in the NST-SS group and ST-S group show a quite close mean CT intensity distribution (*p*-value = 0.232). Among these patients with similar CT performance, those who used steroids therapy (ST-S group) are observed with a better and faster recovery, as shown in CT performance and the shorter average hospitalization time (*p*-value = 0.041). Some previous studies show that chest CT features can be used to assess the severity of pneumonia (38, 39). Therefore, it can be concluded from our study that the CT information is a more important reference for the patients with severe pneumonia when measuring the significance of using steroids therapy compared with the clinical symptoms. Steroid therapy for severe cases whose CT mean intensity is not very high may have more positive effect. We consider that for the patients with really severe CT performance, the hyperinflammatory syndrome may not be the main factor restricting the recovery of the disease, which explains the limited effect of steroids in the ST-SS group.

This study still has some limitations. In total, 3∼5 CT scans were captured for each patient in this work, but not all patients were scanned five times. Since, for some patients, there was a lack of CT scanning at hospitalization admission (CT at cycle 0), and some patients had recovered and discharged from hospital before CT cycle 4. Thus, the CT numbers at each CT cycle are not consistent. Our data volume in patients with significant-severe illness is limited, which may induce some bias in the whole analysis. Finally, our analysis was based on the changes in voxel level of the CT scans and only focused on the amount of change in the relevant focal tissue in each lung lobe. The morphology, distribution, and other characteristics of the lesions were out of consideration.

## Conclusion

In conclusion, our retrospective analysis of patients with COVID-19 showed that the proper use of steroids might significantly promote the recovery of patients with severe illnesses. However, its effect was limited and needed further research to patients with significant-severe illness. CT quantitative results could be used in disease severity evaluation, and compared with clinic symptoms, it may be a better reference for deciding the use of steroids.

## Data Availability Statement

The raw data supporting the conclusions of this article will be made available by the authors, without undue reservation.

## Ethics Statement

The studies involving human participants were reviewed and approved by the Ethics Committee of the Wuhan University People’s Hospital. Written informed consent for participation was not required for this study in accordance with the national legislation and the institutional requirements.

## Author Contributions

All authors listed have made a substantial, direct, and intellectual contribution to the work, and approved it for publication.

## Conflict of Interest

The authors declare that the research was conducted in the absence of any commercial or financial relationships that could be construed as a potential conflict of interest.

## Publisher’s Note

All claims expressed in this article are solely those of the authors and do not necessarily represent those of their affiliated organizations, or those of the publisher, the editors and the reviewers. Any product that may be evaluated in this article, or claim that may be made by its manufacturer, is not guaranteed or endorsed by the publisher.

## Abbreviations

FiO_2_: fraction of inspiration oxygen
IQR: interquartile range
PaO_2_: partial arterial oxygen pressure
SD: standard deviation
RUL: right upper lobe
RML: right middle lobe
RLL: right lower lobe
LUL: left upper lobe
LLL: left lower lobe
HU: Hounsfield unit
ET: emphysema lung tissue
NT: normal lung tissue
PFT: pneumonia fibrotic tissue
GGO: ground-glass opacity
RLO: reticular and linear opacification
SO: consolidations.

## References

[1] World Health Organization [WHO]. *Who Covid-19 Dashboard.* Geneva: World Health Organization (2020).

[2] Stockman LJ Bellamy R Garner P. Sars: systematic review of treatment effects. *PLoS Med.* (2006) 3:e343. 10.1371/journal.pmed.0030343 16968120 PMC1564166

[3] Hui DS. *Systemic Corticosteroid Therapy May Delay Viral Clearance in Patients with Middle East Respiratory Syndrome Coronavirus Infection.* New York, NY: American Thoracic Society (2018). 10.1164/rccm.201712-2371ED 29227752

[4] Covid-19 Treatment Guidelines Panel. *Coronavirus Disease 2019 (Covid-19) Treatment Guidelines. National Institutes of Health.* (2020). Available online at: https://www.covid19treatmentguidelines.nih.gov/ (accessed Septermber 7, 2021). 34003615

[5] Shang L Zhao J Hu Y Du R Cao B. On the use of corticosteroids for 2019-Ncov Pneumonia. *Lancet.* (2020) 395:683–4. 32122468 10.1016/S0140-6736(20)30361-5 PMC7159292

[6] Lee KH Yoon S Jeong GH Kim JY Han YJ Hong SH Efficacy of corticosteroids in patients with sars, mers and Covid-19: a systematic review and meta-analysis. *J Clin Med.* (2020) 9:2392. 10.3390/jcm9082392 PMC7465945 32726951

[7] van Paassen J Vos JS Hoekstra EM Neumann KM Boot PC Arbous SM. Corticosteroid use in Covid-19 patients: a systematic review and meta-analysis on clinical outcomes. *Crit Care.* (2020) 24:1–22. 10.1186/s13054-020-03400-9 33317589 PMC7735177

[8] Yuan M Xu X Xia D Tao Z Yin W Tan W Effects of corticosteroid treatment for non-severe Covid-19 Pneumonia: a propensity score-based analysis. *Shock.* (2020) 54:638–43. 10.1097/SHK.0000000000001574 32496422

[9] Huang R Zhu C Wang J Xue L Li C Yan X Corticosteroid therapy is associated with the delay of Sars-Cov-2 clearance in Covid-19 patients. *Eur J Pharmacol.* (2020) 889:173556. 10.1016/j.ejphar.2020.173556 32941927 PMC7490250

[10] Li Y Xia L. Coronavirus disease 2019 (Covid-19): role of chest ct in diagnosis and management. *Am J Roentgenol.* (2020) 214:1280–6. 10.2214/AJR.20.22954 32130038

[11] Shi H Han X Jiang N Cao Y Alwalid O Gu J Radiological findings from 81 patients with Covid-19 Pneumonia in Wuhan, China: a descriptive study. *Lancet Infect Dis.* (2020) 20:425–34. 10.1016/S1473-3099(20)30086-4 32105637 PMC7159053

[12] Feng Z Yu Q Yao S Luo L Zhou W Mao X Early prediction of disease progression in Covid-19 Pneumonia patients with chest CT and clinical characteristics. *Nat Commun.* (2020) 11:1–9. 10.1038/s41467-020-18786-x 33009413 PMC7532528

[13] Ai T Yang Z Hou H Zhan C Chen C Lv W Correlation of chest CT and RT-PCR testing for coronavirus disease 2019 (Covid-19) in China: a report of 1014 cases. *Radiology.* (2020) 296:E32–40. 10.1148/radiol.2020200642 32101510 PMC7233399

[14] Pan F Ye T Sun P Gui S Liang B Li L Time course of lung changes at chest CT during recovery from coronavirus disease 2019 (Covid-19). *Radiology.* (2020) 295:715–21. 10.1148/radiol.2020200370 32053470 PMC7233367

[15] Yousef HA Moussa EMM Abdel-Razek MZM El-Kholy MMSA Hasan LHS El-Sayed AEA Automated quantification of Covid-19 Pneumonia severity in chest CT using histogram-based multi-level thresholding segmentation. *Egyptian J Radiol Nuclear Med.* (2021) 52:1–10. 10.1186/s43055-021-00602-1

[16] Qiu Y Su Y Tu GW Ju MJ He HY Gu ZY Neutrophil-to-lymphocyte ratio predicts ortality in adult renal transplant recipients with severe community-acquired pneumonia. *Pathogens*. (2020) 9:913. 10.3390/pathogens9110913 PMC7694174 33158161

[17] Lanza E Muglia R Bolengo I Santonocito OG Lisi C Angelotti G Quantitative chest CT analysis in Covid-19 to predict the need for oxygenation support and intubation. *Eur Radiol.* (2020) 30:6770–8. 10.1007/s00330-020-07013-2 32591888 PMC7317888

[18] Chen A Karwoski RA Gierada DS Bartholmai BJ Koo CW. Quantitative CT analysis of diffuse lung disease. *Radiographics.* (2020) 40:28–43. 10.1148/rg.2020190099 31782933

[19] Su Y Ju M-J Ma J-F Tu G-W He H-Y Gu Z-Y Lactate dehydrogenase as a prognostic marker of renal transplant recipients with severe community-acquired pneumonia: a 10-year retrospective study. *Ann Transl Med.* (2019) 7:660. 10.21037/atm.2019.10.75 31930061 PMC6944597

[20] Tu GW Ju MJ Xu M Rong RM He YZ Xue ZG Combination of caspofungin and low-dose trimethoprim/sulfamethoxazole for the treatment of severe Pneumocystis Jirovecii Pneumonia in renal transplant recipients. *Nephrology.* (2013) 18:736–42. 10.1111/nep.12133 24571744

[21] Tu G-W Ju M-J Han Y He H-Y Rong R-M Xu M Moderate-dose glucocorticoids as salvage therapy for severe pneumonia in renal transplant recipients: a single-center feasibility study. *Renal Fail.* (2014) 36:202–9. 10.3109/0886022X.2013.846771 24172054

[22] Tu G-W Ju M-J Zheng Y-J Zhu D-M Xu M Rong R-M An interdisciplinary approach for renal transplant recipients with severe Pneumonia: a single ICU experience. *Intens Care Med.* (2014) 40:914–5. 10.1007/s00134-014-3296-6 24777707 PMC7094935

[23] National Health Commission of the People’s Republic of China. *Diagnosis and Treatment Plan for Covid-19 (Trial Version 6 Revision).* (2020). Available online at: http://www.nhc.gov.cn/yzygj/s7653p/202002/8334a8326dd94d329df351d7da8aefc2/files/b218cfeb1bc54639af227f922bf6b817.pdf (accessed March 16, 2021).

[24] Hofmanninger J Prayer F Pan J Röhrich S Prosch H Langs G. Automatic lung segmentation in routine imaging is primarily a data diversity problem, not a methodology problem. *Eur Radiol Exp.* (2020) 4:50. 10.1186/s41747-020-00173-2 32814998 PMC7438418

[25] Ohkubo H Nakagawa H Niimi A. Computer-based quantitative computed tomography image analysis in idiopathic pulmonary fibrosis: a mini review. *Respiratory Invest.* (2018) 56:5–13. 10.1016/j.resinv.2017.10.003 29325682

[26] Kauczor H-U Heitmann K Heussel CP Marwede D Uthmann T Thelen M. Automatic detection and quantification of ground-glass opacities on high-resolution CT using multiple neural networks: comparison with a density mask. *Am J Roentgenol.* (2000) 175:1329–34. 10.2214/ajr.175.5.1751329 11044035

[27] Shin KE Chung MJ Jung MP Choe BK Lee KS. Quantitative computed tomographic indexes in diffuse interstitial lung disease: correlation with physiologic tests and computed tomography visual scores. *J Comput Assist Tomogr.* (2011) 35:266–71. 10.1097/RCT.0b013e31820ccf18 21412102

[28] De Pascale G Bello G Antonelli M. Steroids in severe Pneumonia: a literature review. *Minerva Anestesiol.* (2011) 77:902–10. 21878872

[29] Póvoa P Salluh JI. What is the role of steroids in Pneumonia therapy? *Curr Opin Infect Dis.* (2012) 25:199–204. 10.1097/QCO.0b013e32834f44c7 22156902

[30] Zha L Li S Pan L Tefsen B Li Y French N Corticosteroid treatment of patients with coronavirus disease 2019 (Covid-19). *Med J Australia.* (2020) 212:416–20. 10.5694/mja2.50577 32266987 PMC7262211

[31] Kolilekas L Loverdos K Giannakaki S Vlassi L Levounets A Zervas E Can steroids reverse the severe Covid-19 induced “Cytokine Storm”? *J Med Virol.* (2020) 92:2866–9. 10.1002/jmv.26165 32530507 PMC7307112

[32] Long C Xu H Shen Q Zhang X Fan B Wang C Diagnosis of the coronavirus disease (Covid-19): rRT-PCR or CT? *Eur J Radiol.* (2020) 126:108961. 10.1016/j.ejrad.2020.108961 32229322 PMC7102545

[33] Jiang Y Guo D Li C Chen T Li R. High-resolution CT features of the Covid-19 infection in Nanchong City: initial and follow-up changes among different clinical types. *Radiol Infect Dis.* (2020) 7:71–7. 10.1016/j.jrid.2020.05.001 32406420 PMC7217764

[34] Yin X Min X Nan Y Feng Z Li B Cai W Assessment of the severity of coronavirus disease: quantitative computed tomography parameters versus semiquantitative visual score. *Korean J Radiol.* (2020) 21:998. 10.3348/kjr.2020.0423 32677384 PMC7369205

[35] Rorat M Jurek T Simon K Guziński M. Value of quantitative analysis in lung computed tomography in patients severely ill with Covid-19. *PLoS One.* (2021) 16:e0251946. 10.1371/journal.pone.0251946 34015025 PMC8136668

[36] Cheng Z Qin L Cao Q Dai J Pan A Yang W Quantitative computed tomography of the coronavirus disease 2019 (Covid-19) Pneumonia. *Radiol Infect Dis.* (2020) 7:55–61. 10.1016/j.jrid.2020.04.004 32346594 PMC7186132

[37] Bartholmai BJ Raghunath S Karwoski RA Moua T Rajagopalan S Maldonado F Quantitative CT imaging of interstitial lung diseases. *J Thoracic Imaging.* (2013) 28:10.1097/RTI.0b013e3182a21969. 10.1097/RTI.0b013e3182a21969 PMC3850512 23966094

[38] Li K Wu J Wu F Guo D Chen L Fang Z The clinical and chest CT features associated with severe and critical Covid-19 Pneumonia. *Invest Radiol.* (2020) 55:327–31. 10.1097/RLI.0000000000000672 32118615 PMC7147273

[39] Ufuk F Demirci M Sagtas E Akbudak IH Ugurlu E Sari T. The prognostic value of Pneumonia severity score and pectoralis muscle area on chest CT in Adult Covid-19 patients. *Eur J Radiol.* (2020) 131:109271. 10.1016/j.ejrad.2020.109271 32942198 PMC7480333

