# Supplementary material for: Steroids Therapy in Patients With Severe COVID-19: Association With Decreasing of Pneumonia Fibrotic Tissue Volume
Source: Front Med (Lausanne). 2022 Jul 14;9:907727. doi: 10.3389/fmed.2022.907727 (PMC9329540; doi:10.3389/fmed.2022.907727)
Supplement: Supplementary file 1 [file Data_Sheet_1.docx]

Supplementary Material

## Supplementary Figures
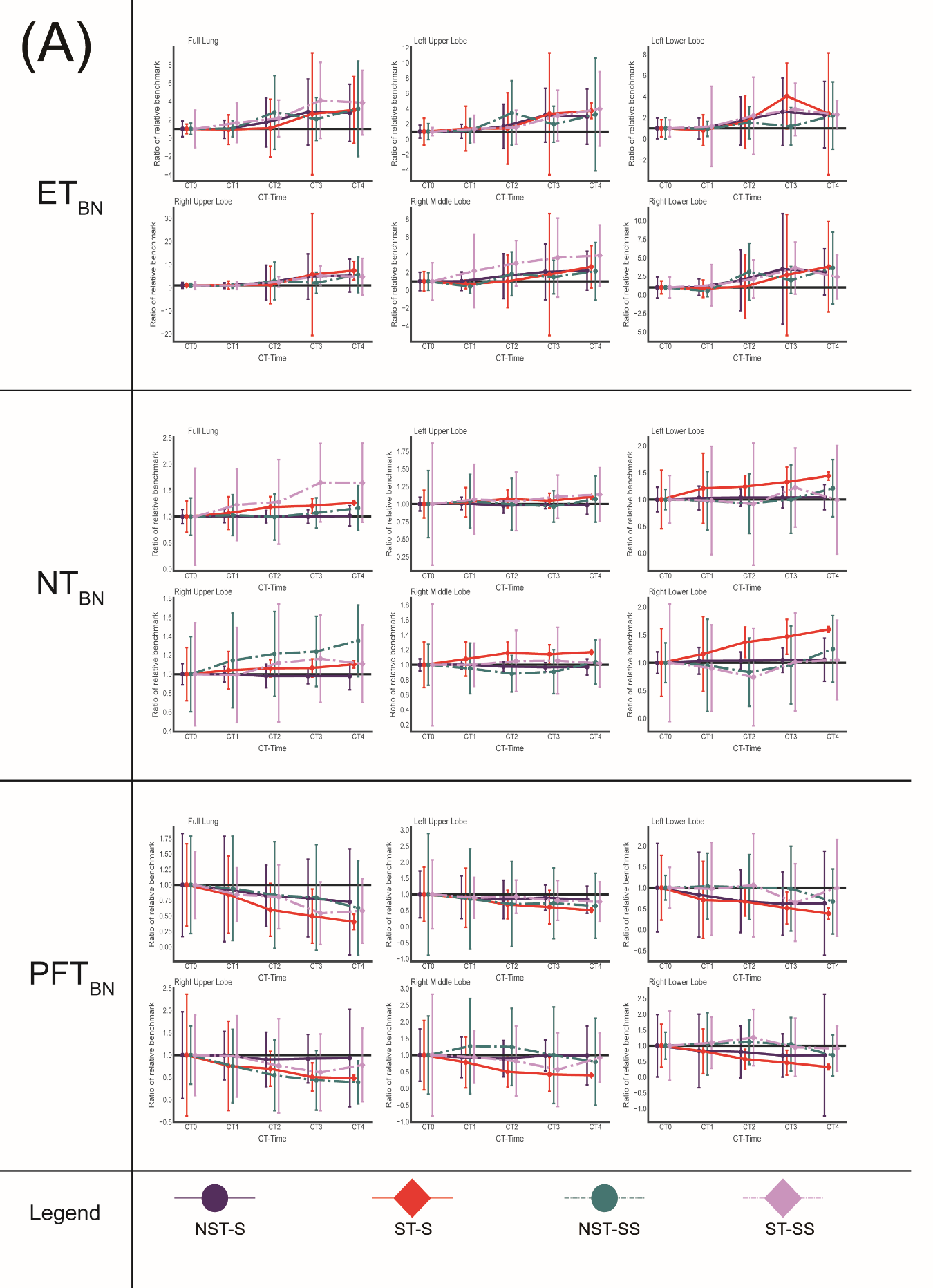


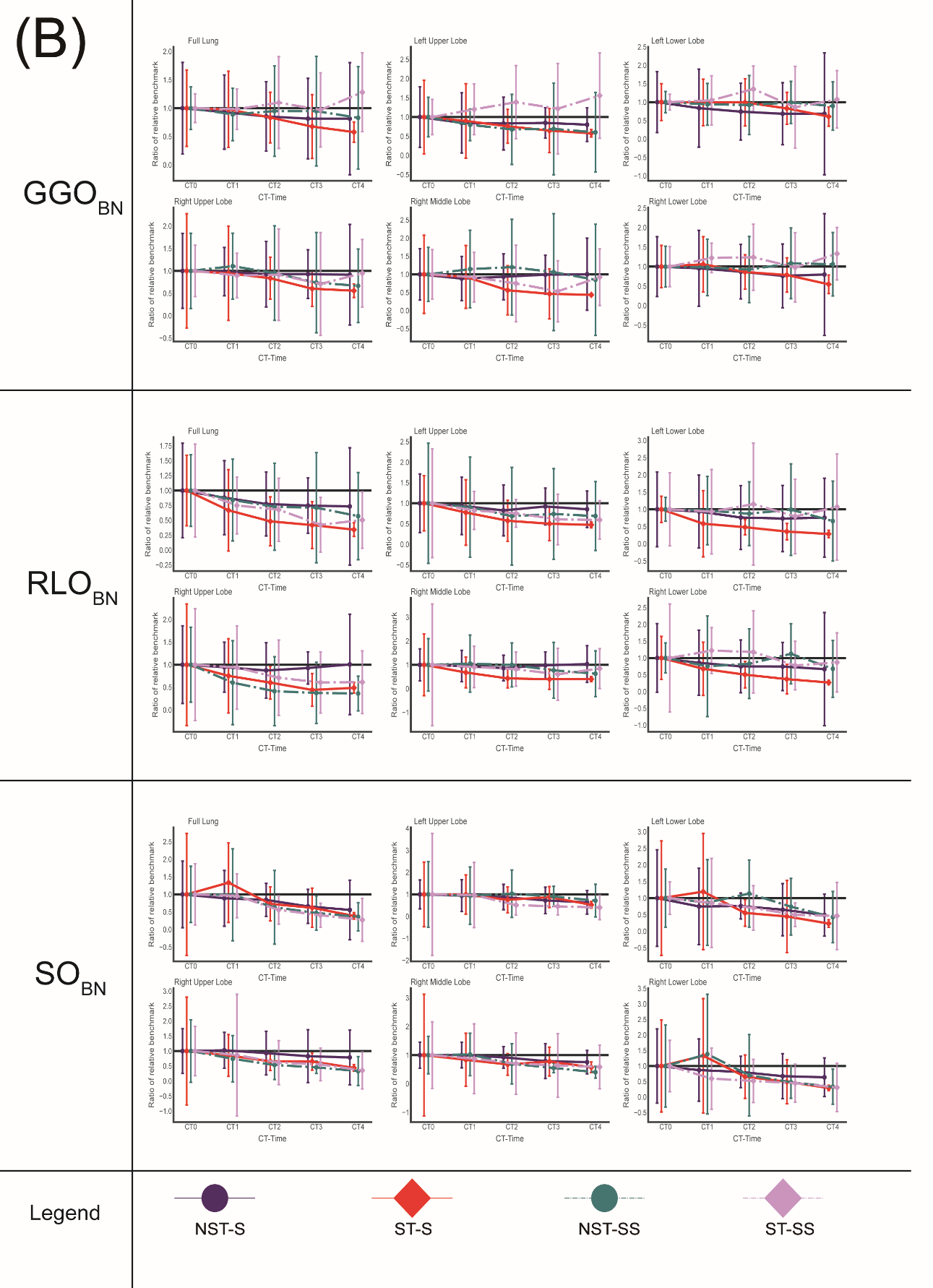


**Supplementary Figure 1. Evolution of baseline normalized (BN) tissue volume along longitudinal CT scanning time points**

Part (A) shows the tissue of ${ET}_{BN}, {NT}_{BN}$, ${PFT}_{BN}$ evolution and Part (B) shows ${GGO}_{BN}, {RLO}_{BN}$, ${SO}_{BN}$ evolution respectively. The normalized tissue is defined as： ${Tissue}_{BN\_{CT}_{i}}=\frac{Tissue volume at {CT}_{i}in ROI}{Tissue volume of ROI at {CT}_{0}}$ where ${CT}_{i}=\left\{ {CT}_{0},\cdots,{CT}_{4} \right\}, Tissue=\left\{ ET,NT,GGO,RLO,SO \right\}$, and $ROI=\left\{ \mathrm{Full} Lung, LUL, LLL, RUL, RML, RLL \right\}$. ET: emphysema lung tissue; NT: normal lung tissue; PFT: pneumonia fibrotic tissue; GGO: ground-glass opacity; RLO: reticular and linear opacification; SO: consolidations; NST-S: non-steroids-treated group with severe illness; ST-S: steroids-treated group with severe illness; NST-SS: non-steroids-treated group with significant-severe illness; ST-SS: steroids-treated group with significant-severe illness.

## Supplementary Tables

| **Lung Lobe** | **CT Sequence** | **CT_0_** | | **CT_1_** | | **CT_2_** | | **CT_3_** | | **CT_4_** | |
| --- | --- | --- | --- | --- | --- | --- | --- | --- | --- | --- | --- |
|  | **Patient Category** | **NST-S** | **ST-S** | **NST-S** | **ST-S** | **NST-S** | **ST-S** | **NST-S** | **ST-S** | **NST-S** | **ST-S** |
|  | **Patient Number** | **46** | **10** | **46** | **12** | **40** | **12** | **27** | **12** | **16** | **9** |
| **Full Lung** | $\boldsymbol{\%}\mathbf{ET}$ | **1.15**  **[0.97]** | **0.48**  **[0.25] ^**^** | **1.10**  **[1.12]** | **0.44**  **[0.77] ^**^** | **1.96**  **[3.07]** | **0.52**  **[1.51] ^*^** | **3.26**  **[4.14]** | **1.27**  **[3.19]** | **3.16**  **[3.60]** | **1.47**  **[1.75] ^*^** |
|  | $\boldsymbol{\%}\mathbf{NT}$ | **84.46 [11.76]** | **68.56**  **[20.49] ^**^** | **85.56 [10.76]** | **73.19 [21.42] ^**^** | **84.51 [10.01]** | **81.10**  **[13.61] ^*^** | **84.51**  **[11.17]** | **82.74**  **[9.72]** | **85.64 [16.27]** | **86.6**  **[2.12]** |
|  | $\boldsymbol{\%}\mathbf{PFT}$ | **13.67 [11.36]** | **31.06**  **[20.65] ^**^** | **12.76 [11.62]** | **26.16 [19.35] ^**^** | **11.27 [6.77]** | **18.55**  **[13.23] ^**^** | **10.78**  **[8.56]** | **15.42**  **[13.60] ^*^** | **9.91 [11.73]** | **12.49**  **[3.85]** |
|  | $\boldsymbol{\%}\mathbf{GGO}$ | **6.28**  **[5.06]** | **11.63**  **[7.83] ^*^** | **5.85**  **[4.11]** | **11.40**  **[7.82] ^**^** | **5.37**  **[3.84]** | **9.71**  **[5.18] ^**^** | **5.13**  **[4.46]** | **7.87**  **[6.53] ^*^** | **5.12**  **[6.20]** | **6.73**  **[2.09]** |
|  | $\boldsymbol{\%}\mathbf{RLO}$ | **5.34**  **[4.24]** | **13.60**  **[8.06] ^**^** | **4.70**  **[3.32]** | **9.09**  **[9.3] ^**^** | **4.14**  **[2.87]** | **6.57**  **[5.57] ^**^** | **3.99**  **[2.48]** | **5.72**  **[5.34] ^*^** | **3.92**  **[5.25]** | **4.67**  **[1.56]** |
|  | $\boldsymbol{\%}\mathbf{SO}$ | **1.90**  **[1.80]** | **3.23**  **[5.6] ^*^** | **1.69**  **[1.51]** | **4.31**  **[3.66] ^**^** | **1.6**  **[0.89]** | **2.36**  **[1.57] ^**^** | **1.25**  **[0.92]** | **2.00**  **[1.81] ^*^** | **1.06**  **[1.61]** | **1.25**  **[0.3]** |
| **Left Upper Lobe** | $\boldsymbol{\%}\mathbf{ET}$ | **1.56**  **[1.29]** | **0.63**  **[1.1] ^*^** | **1.64**  **[1.39]** | **0.88**  **[1.84] ^*^** | **2.62**  **[4.54]** | **0.89**  **[2.95] ^*^** | **4.90**  **[5.5]** | **2.10**  **[5.02] ^*^** | **4.64**  **[5.65]** | **2.35**  **[0.63] ^*^** |
|  | $\boldsymbol{\%}\mathbf{NT}$ | **86.72**  **[8.42]** | **79.88**  **[15.71]** | **87.58 [7.59]** | **81.89 [16.82] ^*^** | **84.72 [9.16]** | **85.93**  **[10.12]** | **85.36**  **[4.25]** | **83.88**  **[8.25]** | **85.49 [11.68]** | **87.96**  **[1.69] ^*^** |
|  | $\boldsymbol{\%}\mathbf{PFT}$ | **10.74**  **[7.79]** | **19.49**  **[16.45]** | **9.81**  **[7.12]** | **17.43 [17.88] ^**^** | **9.04**  **[6.38]** | **13.32**  **[8.61] ^*^** | **9.64**  **[4.25]** | **11.78**  **[10.12]** | **8.94**  **[4.53]** | **9.87**  **[1.34]** |
|  | $\boldsymbol{\%}\mathbf{GGO}$ | **5.42**  **[4.27]** | **9.04**  **[8.68]** | **4.60**  **[4.25]** | **8.12**  **[8.78] ^**^** | **4.50**  **[3.73]** | **6.86**  **[4.00] ^*^** | **4.60**  **[2.15]** | **5.84**  **[5.17]** | **4.34**  **[2.37]** | **5.20**  **[0.86]** |
|  | $\boldsymbol{\%}\mathbf{RLO}$ | **4.09**  **[2.92]** | **8.44**  **[5.66]** | **3.84**  **[2.86]** | **6.54**  **[6.73] ^**^** | **3.38**  **[2.53]** | **4.85**  **[4.22] ^*^** | **3.77**  **[1.83]** | **4.24**  **[3.46]** | **3.49**  **[1.82]** | **4.00**  **[0.61]** |
|  | $\boldsymbol{\%}\mathbf{SO}$ | **1.25**  **[0.82]** | **1.75**  **[2.57]** | **1.18**  **[0.90]** | **1.74**  **[1.56] ^*^** | **1.10**  **[0.72]** | **1.32**  **[1.03]** | **0.91**  **[0.75]** | **1.54**  **[0.83]** | **0.80**  **[0.67]** | **0.92**  **[0.26]** |
| **Left Lower Lobe** | $\boldsymbol{\%}\mathbf{ET}$ | **1.06**  **[1.04]** | **0.43**  **[0.35] ^**^** | **1.04**  **[1.06]** | **0.35**  **[0.63] ^**^** | **1.78**  **[2.42]** | **0.68**  **[1.08] ^**^** | **2.71**  **[3.4]** | **1.74**  **[1.34] ^**^** | **2.42**  **[3.34]** | **1.02**  **[2.48]** |
|  | $\boldsymbol{\%}\mathbf{NT}$ | **80.49**  **[18.3]** | **58.00**  **[31.66] ^**^** | **82.64 [17.73]** | **70.00**  **[37.98] ^**^** | **83.32 [13.11]** | **71.92 [11.77] ^**^** | **83.42**  **[15.44]** | **76.84**  **[16.04]** | **83.98 [18.77]** | **83.37**  **[4.45]** |
|  | $\boldsymbol{\%}\mathbf{PFT}$ | **18.64 [19.62]** | **41.56**  **[32.0] ^**^** | **15.52 [18.82]** | **29.48 [38.14] ^**^** | **12.75 [13.96]** | **27.77 [14.18] ^**^** | **11.54**  **[14.07]** | **21.44**  **[15.92] ^*^** | **11.75 [23.12]** | **15.94**  **[5.65]** |
|  | $\boldsymbol{\%}\mathbf{GGO}$ | **8.72**  **[7.21]** | **14.88**  **[7.45] ^*^** | **7.29**  **[9.22]** | **14.77**  **[9.38] ^**^** | **6.46**  **[6.72]** | **14.76**  **[9.51] ^**^** | **5.96**  **[7.33]** | **12.33**  **[6.47] ^**^** | **5.95 [14.41]** | **9.05**  **[3.88]** |
|  | $\boldsymbol{\%}\mathbf{RLO}$ | **6.30**  **[6.84]** | **20.29**  **[7.70] ^**^** | **5.90**  **[6.66]** | **11.82 [19.54] ^**^** | **4.80**  **[5.83]** | **9.76**  **[4.53] ^**^** | **4.62**  **[6.05]** | **7.20**  **[4.8] ^*^** | **4.76**  **[7.22]** | **5.76**  **[2.16]** |
|  | $\boldsymbol{\%}\mathbf{SO}$ | **2.38**  **[3.46]** | **5.78**  **[9.96] ^**^** | **1.78**  **[2.74]** | **6.90**  **[10.13] ^**^** | **1.81**  **[0.93]** | **3.20**  **[2.32] ^**^** | **1.55**  **[1.87]** | **2.58**  **[6.27] ^*^** | **1.16**  **[1.51]** | **1.35**  **[0.67]** |
| **Right Upper Lobe** | $\boldsymbol{\%}\mathbf{ET}$ | **0.54**  **[0.67]** | **0.20**  **[0.10] ^**^** | **0.50**  **[0.65]** | **0.22**  **[0.35] ^*^** | **1.20**  **[4.08]** | **0.23**  **[1.61] ^*^** | **2.62**  **[5.30]** | **1.14**  **[5.27]** | **2.82**  **[3.84]** | **1.48**  **[0.82]** |
|  | $\boldsymbol{\%}\mathbf{NT}$ | **89.44**  **[10.00]** | **81.28**  **[22.85]** | **89.42 [7.17]** | **84.62**  **[16.1] ^*^** | **87.68 [11.02]** | **86.42**  **[7.44]** | **87.77**  **[7.39]** | **86.66**  **[7.72]** | **87.74 [12.95]** | **89.79**  **[3.21]** |
|  | $\boldsymbol{\%}\mathbf{PFT}$ | **10.00**  **[9.73]** | **18.52**  **[25.28]** | **9.91**  **[5.41]** | **14.04 [18.56] ^*^** | **9.04**  **[6.09]** | **12.88**  **[7.18]** | **9.21**  **[5.45]** | **9.41**  **[5.84]** | **9.36 [10.91]** | **8.89**  **[1.25]** |
|  | $\boldsymbol{\%}\mathbf{GGO}$ | **5.16**  **[4.31]** | **8.13**  **[10.39]** | **5.07**  **[2.78]** | **7.70**  **[8.55] ^*^** | **4.78**  **[3.77]** | **6.80**  **[3.80]** | **4.79**  **[2.80]** | **4.90**  **[3.22]** | **4.72**  **[5.79]** | **4.53**  **[1.23]** |
|  | $\boldsymbol{\%}\mathbf{RLO}$ | **3.69**  **[3.17]** | **7.14**  **[9.60] ^*^** | **3.49**  **[2.04]** | **5.38**  **[5.86] ^*^** | **3.22**  **[2.27]** | **4.31**  **[2.60]** | **3.42**  **[1.31]** | **3.16**  **[2.58]** | **3.71**  **[4.10]** | **3.48**  **[0.84]** |
|  | $\boldsymbol{\%}\mathbf{SO}$ | **1.17**  **[0.87]** | **2.01**  **[3.62]** | **1.20**  **[0.70]** | **1.72**  **[1.40]** | **1.09**  **[0.85]** | **1.32**  **[0.62]** | **0.97**  **[1.03]** | **1.31**  **[0.58]** | **0.92**  **[1.07]** | **0.88**  **[0.22]** |
| **Right Middle Lobe** | $\boldsymbol{\%}\mathbf{ET}$ | **1.72**  **[1.78]** | **0.62**  **[0.68] ^**^** | **1.76**  **[1.79]** | **0.42**  **[0.3] ^**^** | **2.74**  **[4.34]** | **0.63**  **[1.85] ^**^** | **3.58**  **[5.41]** | **1.10**  **[4.25] ^*^** | **3.80**  **[3.75]** | **1.64**  **[1.48] ^*^** |
|  | $\boldsymbol{\%}\mathbf{NT}$ | **89.27**  **[7.20]** | **75.12**  **[22.91] *** | **89.50**  **[4.98]** | **81.00**  **[17.25] ^**^** | **87.10**  **[5.94]** | **86.96**  **[10.95]** | **86.37**  **[5.38]** | **85.72**  **[9.50]** | **86.66 [9.69]** | **87.85**  **[2.12]** |
|  | $\boldsymbol{\%}\mathbf{PFT}$ | **8.86**  **[6.95]** | **23.96**  **[24.93] *** | **8.30**  **[5.4]** | **18.74 [18.28] ^**^** | **7.93**  **[4.83]** | **11.96**  **[10.87] ^**^** | **8.75**  **[4.14]** | **10.14**  **[12.32] ^*^** | **8.75**  **[7.84]** | **9.50**  **[0.69]** |
|  | $\boldsymbol{\%}\mathbf{GGO}$ | **4.27**  **[3.03]** | **11.18**  **[12.06] *** | **3.77**  **[2.60]** | **10.40**  **[9.69] ^**^** | **3.98**  **[2.71]** | **6.26**  **[7.59] ^**^** | **4.23**  **[2.29]** | **5.16**  **[7.73] ^*^** | **4.28**  **[4.25]** | **4.85**  **[0.36]** |
|  | $\boldsymbol{\%}\mathbf{RLO}$ | **3.35**  **[2.25]** | **9.21**  **[11.94] *** | **3.18**  **[2.21]** | **6.24**  **[5.92] ^**^** | **2.90**  **[1.80]** | **3.96**  **[3.70] ^**^** | **3.28**  **[1.87]** | **3.64**  **[3.94]** | **3.45**  **[2.59]** | **3.68**  **[1.00]** |
|  | $\boldsymbol{\%}\mathbf{SO}$ | **1.30**  **[0.59]** | **1.96**  **[4.17] *** | **1.30**  **[0.58]** | **1.64**  **[1.82] ^*^** | **1.20**  **[0.49]** | **1.32**  **[0.74]** | **1.03**  **[0.79]** | **1.52**  **[0.99] ^*^** | **0.98**  **[0.53]** | **1.13**  **[0.36]** |
| **Right Lower Lobe** | $\boldsymbol{\%}\mathbf{ET}$ | **0.53**  **[0.76]** | **0.23**  **[0.20] ^**^** | **0.55**  **[0.60]** | **0.19**  **[0.27] ^**^** | **1.05**  **[2.18]** | **0.26**  **[0.99] ^**^** | **1.85**  **[3.96]** | **0.62**  **[1.88] ^*^** | **1.64**  **[1.65]** | **0.87**  **[1.40]** |
|  | $\boldsymbol{\%}\mathbf{NT}$ | **80.44 [15.73]** | **52.94**  **[32.08] ^**^** | **83.26 [19.26]** | **61.26 [35.65] ^**^** | **83.52 [12.55]** | **72.50**  **[14.53] ^**^** | **84.31**  **[17.74]** | **77.63**  **[16.59] ^*^** | **84.71 [31.27]** | **84.65**  **[2.65]** |
|  | $\boldsymbol{\%}\mathbf{PFT}$ | **16.9**  **[16.79]** | **46.79**  **[32.00] ^**^** | **14.12 [19.75]** | **38.50**  **[33.41] ^**^** | **13.57 [13.94]** | **26.86**  **[14.76] ^**^** | **11.62**  **[11.58]** | **21.58**  **[18.35] ^**^** | **11.84 [32.74]** | **14.99**  **[4.01]** |
|  | $\boldsymbol{\%}\mathbf{GGO}$ | **7.68**  **[5.89]** | **14.80**  **[8.03] *** | **7.34**  **[7.45]** | **15.68 [10.47] ^**^** | **6.68**  **[5.35]** | **12.75**  **[6.54] ^**^** | **5.86**  **[6.27]** | **11.62**  **[6.52] ^**^** | **6.12 [11.98]** | **8.09**  **[3.51]** |
|  | $\boldsymbol{\%}\mathbf{RLO}$ | **6.16**  **[6.29]** | **19.23**  **[12.34] ^**^** | **5.28**  **[5.98]** | **13.01 [15.27] ^**^** | **4.62**  **[4.84]** | **9.68**  **[7.61] ^**^** | **4.59**  **[4.42]** | **7.12**  **[8.46] ^*^** | **4.11 [10.38]** | **5.17**  **[1.24]** |
|  | $\boldsymbol{\%}\mathbf{SO}$ | **2.20**  **[2.63]** | **5.94**  **[8.81] ^**^** | **1.91**  **[2.21]** | **7.91**  **[10.95] ^**^** | **1.78**  **[1.11]** | **3.89**  **[4.16] ^**^** | **1.48**  **[1.59]** | **2.92**  **[4.21] ^**^** | **1.40**  **[1.37]** | **1.73**  **[0.51]** |

**Supplementary Table1. CT quantitative results progress over time in the patients with severe illness**

For CT quantitative results, normally distributed groups were shown as mean [SD], and non-normally distributed groups were shown as median [IQR]. CT quantitative results were expressed in the form of volume percentage related to the full lung and each lung lobe. SD: standard deviation; IQR: interquartile range; NST-S: non-steroids-treated group with severe illness; ST-S: steroids-treated group with severe illness; ET: emphysema lung tissue; NT: normal lung tissue; PFT: pneumonia fibrotic tissue; GGO: ground-glass opacity; RLO: reticular and linear opacification; SO: consolidations. * donates P<0.05 and ** donates P<0.01 between two groups at each CT scan.

| **Lung Region** | **CT Sequence** | **CT_0_** | | **CT_1_** | | **CT_2_** | | **CT_3_** | | **CT_4_** | |
| --- | --- | --- | --- | --- | --- | --- | --- | --- | --- | --- | --- |
|  | **Patient Category** | **NST-SS** | **ST-SS** | **NST-SS** | **ST-SS** | **NST-SS** | **ST-SS** | **NST-SS** | **ST-SS** | **NST-SS** | **ST-SS** |
|  | **Patient Number** | **7** | **7** | **7** | **7** | **6** | **13** | **6** | **12** | **6** | **10** |
| **Full Lung** | $\boldsymbol{\%}\mathbf{ET}$ | **0.62**  **[0.39]** | **0.65**  **[1.33]** | **0.64 [0.55]** | **1.08**  **[1.40]** | **1.74 [2.49]** | **1.39**  **[1.30]** | **1.29 [1.45]** | **2.67**  **[2.69]** | **1.98 [3.23]** | **2.51 [2.29]** |
|  | $\boldsymbol{\%}\mathbf{NT}$ | **67.51 [23.96]** | **37.26 [34.35]** | **69.45 [26.42]** | **45.58 [25.34]** | **67.06 [29.7]** | **47.67 [30.05]** | **72.19 [19.49]** | **61.38 [27.89]** | **78.46 [28.87]** | **61.26 [28.14]** |
|  | $\boldsymbol{\%}\mathbf{PFT}$ | **31.86 [25.06]** | **62.40**  **[33.71]** | **30.07 [26.85]** | **52.54 [27.11]** | **26.64 [27.47]** | **50.49 [32.24]** | **25.39 [27.25]** | **33.81 [31.49]** | **20.02 [24.37]** | **36.24 [32.61]** |
|  | $\boldsymbol{\%}\mathbf{GGO}$ | **13.53 [5.09]** | **14.19 [**  **3.58]** | **12.05 [6.27]** | **13.95**  **[5.08]** | **12.84 [10.79]** | **15.59 [11.48]** | **12.84 [13.08]** | **13.77**  **[9.28]** | **11.24 [12.22]** | **18.18 [9.84]** |
|  | $\boldsymbol{\%}\mathbf{RLO}$ | **13.40**  **[8.06]** | **27.59 [21.50]** | **11.27 [9.20]** | **20.69 [13.12]** | **9.75 [9.77]** | **18.69 [14.56] ^*^** | **9.54 [12.38]** | **11.75 [12.71]** | **7.65 [9.8]** | **13.94 [13.1]** |
|  | $\boldsymbol{\%}\mathbf{SO}$ | **6.89**  **[5.51]** | **14.13 [12.34]** | **6.80 [9.02]** | **13.55**  **[8.84]** | **4.36 [7.23]** | **7.88**  **[5.75]** | **3.24 [3.36]** | **5.74**  **[4.87] ^*^** | **2.50 [2.75]** | **3.87 [8.65]** |
| **Left Upper Lobe** | $\boldsymbol{\%}\mathbf{ET}$ | **0.85**  **[0.88]** | **0.94 [0.49]** | **0.95 [1.35]** | **1.28**  **[1.68]** | **2.92 [3.59]** | **1.48**  **[2.09]** | **1.66 [2.01]** | **2.91**  **[3.13]** | **2.77 [6.28]** | **3.73 [4.58]** |
|  | $\boldsymbol{\%}\mathbf{NT}$ | **78.57 [37.44]** | **54.75 [47.47]** | **81.83 [29.92]** | **58.48 [27.18] ^*^** | **78.28 [29.08]** | **56.88 [22.82]** | **75.94 [17.6]** | **60.64 [16.73]** | **84.3 [25.92]** | **62.17 [20.92]** |
|  | $\boldsymbol{\%}\mathbf{PFT}$ | **20.04 [37.88]** | **44.23 [47.24]** | **17.22 [31.28]** | **39.53 [28.07]** | **14.02 [26.43]** | **41.09 [22.88]** | **14.50 [21.95]** | **35.86 [27.41]** | **12.90 [20.22]** | **34.06 [27.36]** |
|  | $\boldsymbol{\%}\mathbf{GGO}$ | **10.75 [5.53]** | **11.71 [5.40]** | **8.57 [4.44]** | **14.06**  **[7.74]** | **7.29 [9.83]** | **16.25 [11.16]** | **7.43 [12.83]** | **14.25 [13.81]** | **6.46 [11.16]** | **18.25 [13.02]** |
|  | $\boldsymbol{\%}\mathbf{RLO}$ | **7.46**  **[10.90]** | **21.97 [29.02] ^*^** | **6.75 [9.10]** | **18.13 [10.18] ^*^** | **5.12 [8.86]** | **16.58**  **[7.40] ^*^** | **5.52 [8.23]** | **13.31 [13.52]** | **5.14 [6.25]** | **13.07 [10.27]** |
|  | $\boldsymbol{\%}\mathbf{SO}$ | **2.01**  **[3.01]** | **6.85 [18.94]** | **1.89 [2.62]** | **6.64**  **[10.13]** | **2.07 [2.16]** | **3.51**  **[3.08]** | **1.82 [0.92]** | **3.12**  **[2.66] ^*^** | **1.44 [1.49]** | **2.78 [3.81]** |
| **Left Lower Lobe** | $\boldsymbol{\%}\mathbf{ET}$ | **0.67**  **[0.68]** | **0.66 [0.43]** | **0.63 [0.47]** | **0.78**  **[2.51]** | **1.03 [1.02]** | **1.44**  **[2.42]** | **0.79 [1.19]** | **1.84**  **[1.64] ^*^** | **1.48 [2.13]** | **1.51**  **[0.90]** |
|  | $\boldsymbol{\%}\mathbf{NT}$ | **58.83 [11.05]** | **51.34 [22.87]** | **57.45 [32.09]** | **50.30**  **[51.76]** | **54.24 [32.72]** | **46.90**  **[58.24]** | **58.94 [37.39]** | **62.70**  **[37.77]** | **71.11 [31.35]** | **50.96 [51.88]** |
|  | $\boldsymbol{\%}\mathbf{PFT}$ | **40.68 [11.96]** | **47.94 [23.27]** | **42.15 [31.98]** | **46.67 [53.18]** | **41.21 [31.55]** | **50.86 [59.09]** | **39.78 [41.10]** | **31.00**  **[44.23]** | **27.43 [31.5]** | **47.75 [55.26]** |
|  | $\boldsymbol{\%}\mathbf{GGO}$ | **17.79 [5.15]** | **13.26 [2.85]** | **16.70 [10.10]** | **13.91**  **[8.85]** | **16.40 [14.25]** | **17.96**  **[8.31]** | **17.63 [10.18]** | **11.41**  **[14.70]** | **15.88 [11.64]** | **14.23 [10.34]** |
|  | $\boldsymbol{\%}\mathbf{RLO}$ | **16.10**  **[5.58]** | **16.50 [17.51]** | **15.54 [15.99]** | **15.38 [20.22]** | **14.06 [14.88]** | **19.01 [29.17]** | **15.96 [21.40]** | **13.10**  **[17.85]** | **10.61 [18.68]** | **17.65 [25.45]** |
|  | $\boldsymbol{\%}\mathbf{SO}$ | **8.50**  **[7.43]** | **14.66 [7.14]** | **7.36 [10.98]** | **12.52 [19.69]** | **9.66 [8.61]** | **10.33**  **[9.80]** | **6.29 [7.25]** | **7.25**  **[5.23]** | **3.63 [6.55]** | **6.77 [14.84]** |
| **Right Upper Lobe** | $\boldsymbol{\%}\mathbf{ET}$ | **0.38**  **[0.32]** | **0.46 [0.81]** | **0.27 [0.36]** | **0.44**  **[0.80]** | **1.12 [3.09]** | **1.16**  **[1.04]** | **0.80 [1.76]** | **2.40**  **[1.95]** | **2.13 [2.94]** | **2.18 [3.65]** |
|  | $\boldsymbol{\%}\mathbf{NT}$ | **62.02 [24.64]** | **62.17 [33.77]** | **71.13 [31.03]** | **61.65 [31.22]** | **75.32 [27.78]** | **69.56 [38.73]** | **76.96 [22.94]** | **72.34 [28.72]** | **83.90 [23.48]** | **69.00 [25.52]** |
|  | $\boldsymbol{\%}\mathbf{PFT}$ | **37.86 [24.76]** | **37.25 [33.70]** | **28.59 [31.14]** | **36.23**  **[33.90]** | **20.70 [30.05]** | **28.25 [39.46]** | **16.46 [25.35]** | **22.82 [31.99]** | **14.79 [18.26]** | **28.98 [30.60]** |
|  | $\boldsymbol{\%}\mathbf{GGO}$ | **13.31 [11.17]** | **15.76 [9.05]** | **14.71 [9.80]** | **14.14**  **[7.83]** | **12.66 [14.04]** | **14.40**  **[16.10]** | **9.83 [14.90]** | **11.16**  **[18.10]** | **8.88 [10.91]** | **14.92 [11.89]** |
|  | $\boldsymbol{\%}\mathbf{RLO}$ | **15.47 [12.82]** | **15.53 [19.22]** | **9.32 [14.35]** | **14.55**  **[14.30]** | **6.40 [11.81]** | **11.03 [12.95]** | **5.81 [10.51]** | **9.43**  **[10.53]** | **5.62 [5.94]** | **9.53 [10.78]** |
|  | $\boldsymbol{\%}\mathbf{SO}$ | **3.92**  **[4.08]** | **5.68 [4.67]** | **2.91 [3.03]** | **4.91**  **[11.53]** | **2.10**  **[1.90]** | **3.50**  **[4.17]** | **1.79 [0.81]** | **3.16**  **[3.09]** | **1.30 [1.89]** | **2.00**  **[3.49]** |
| **Right Middle Lobe** | $\boldsymbol{\%}\mathbf{ET}$ | **1.37**  **[1.36]** | **0.98 [2.07]** | **0.57 [1.08]** | **2.14**  **[4.06]** | **2.54 [3.38]** | **2.98**  **[2.51]** | **2.04 [2.59]** | **3.61**  **[4.36]** | **2.94 [4.45]** | **3.84 [3.37]** |
|  | $\boldsymbol{\%}\mathbf{NT}$ | **80.23 [22.0]** | **68.44 [55.64]** | **76.26 [27.18]** | **68.50**  **[19.73]** | **70.66 [19.55]** | **71.90**  **[27.94]** | **72.82 [23.63]** | **72.23 [30.46]** | **83.15 [23.66]** | **69.76 [21.59]** |
|  | $\boldsymbol{\%}\mathbf{PFT}$ | **18.35 [21.54]** | **30.43 [55.64]** | **23.30 [26.24]** | **28.61 [23.82]** | **22.84 [21.27]** | **24.95 [31.88]** | **18.3 [26.59]** | **17.26 [33.66]** | **14.68 [23.96]** | **27.99 [22.47]** |
|  | $\boldsymbol{\%}\mathbf{GGO}$ | **10.03 [7.48]** | **15.37 [10.51]** | **11.48 [10.75]** | **14.14 [10.54]** | **12.02 [13.24]** | **11.5**  **[16.26]** | **10.64 [16.19]** | **8.18**  **[12.97]** | **8.51 [15.39]** | **14.18 [12.00]** |
|  | $\boldsymbol{\%}\mathbf{RLO}$ | **8.62**  **[9.49]** | **12.20 [31.21]** | **9.07 [10.33]** | **11.09 [10.61]** | **8.59 [7.97]** | **9.98**  **[8.74]** | **6.63 [10.13]** | **7.35**  **[13.29]** | **5.41 [8.28]** | **10.28 [10.24]** |
|  | $\boldsymbol{\%}\mathbf{SO}$ | **3.21**  **[2.10]** | **3.39 [3.95]** | **3.28 [2.38]** | **2.95**  **[4.14]** | **2.23 [2.26]** | **2.39**  **[3.64]** | **1.77 [0.53]** | **2.17**  **[3.77]** | **1.29 [0.66]** | **1.99 [2.59]** |
| **Right Lower Lobe** | $\boldsymbol{\%}\mathbf{ET}$ | **0.35**  **[0.09]** | **0.51 [0.72]** | **0.18 [0.27]** | **0.66**  **[1.42]** | **1.09 [1.35]** | **1.09**  **[1.31]** | **0.70 [0.64]** | **1.84**  **[1.79]** | **1.27 [1.70]** | **1.23 [1.51]** |
|  | $\boldsymbol{\%}\mathbf{NT}$ | **54.60 [19.43]** | **50.51 [53.37]** | **52.04 [45.27]** | **45.55 [39.31]** | **45.36 [33.45]** | **37.37 [43.97]** | **52.43 [38.28]** | **51.24 [44.51]** | **68.1 [32.66]** | **53.12 [36.01]** |
|  | $\boldsymbol{\%}\mathbf{PFT}$ | **45.06 [19.30]** | **48.98 [54.09]** | **47.78 [44.67]** | **53.60**  **[39.96]** | **50.15 [32.03]** | **61.57 [43.64]** | **47.03 [38.37]** | **46.52 [45.38]** | **31.24 [29.36]** | **44.67 [34.99]** |
|  | $\boldsymbol{\%}\mathbf{GGO}$ | **16.80**  **[8.78]** | **14.23 [7.31]** | **16.46 [12.15]** | **17.40**  **[5.37]** | **15.54 [14.22]** | **17.65 [12.04]** | **18.22 [15.22]** | **13.94 [12.63]** | **17.83 [13.66]** | **18.94 [9.56]** |
|  | $\boldsymbol{\%}\mathbf{RLO}$ | **16.61 [7.42]** | **20.41 [32.86]** | **12.51 [24.88]** | **24.92 [13.94]** | **13.77 [17.21]** | **24.04 [24.97]** | **18.67 [14.89]** | **15.82 [14.79]** | **11.26 [14.08]** | **17.72 [17.95]** |
|  | $\boldsymbol{\%}\mathbf{SO}$ | **11.64 [15.41]** | **21.60 [17.88]** | **16.11 [22.47]** | **12.82 [21.38]** | **8.17 [15.29]** | **11.19 [15.03]** | **5.54 [6.03]** | **9.66**  **[13.23]** | **3.85 [6.59]** | **6.62 [16.81]** |

**Supplementary Table 2. CT quantitative results progress over time of the patients with significant severe illness** For CT quantitative results, normally distributed groups were shown as mean [SD], and non-normally distributed groups were shown as median [IQR]. CT quantitative results were expressed in the form of volume percentage related to the full lung and each lung lobe. SD: standard deviation; IQR: interquartile range; NST-SS: non-steroids-treated group with significant-severe illness; ST-SS: steroids-treated group with significant-severe illness; ET: emphysema lung tissue; NT: normal lung tissue; PFT: pneumonia fibrotic tissue; GGO: ground-glass opacity; RLO: reticular and linear opacification; SO: consolidations. * donates P<0.05 and ** donates P<0.01 between two groups at each CT scan.
